# Endoscopic transorbital approaches: a video compendium

**DOI:** 10.3171/2025.1.FOCVID24142

**Published:** 2025-04-01

**Authors:** Theodore H. Schwartz, Walavan Sivakumar, Doo-Sik Kong, Darlene Lubbe, Matteo De Notaris, Alberto Di Somma, Kris S. Moe

**Affiliations:** 1 Scarsdale, New York;; 2 Department of Neurosciences, Pacific Neurosciences Institute, Santa Monica, California;; 3 Department of Neurosurgery, Samsung Medical Center, Seoul, South Korea;; 4 Department of Otolaryngology, Groote Schuur Hospital, Cape Town, South Africa;; 5 Department of Neurosurgery, University of Salerno, Italy;; 6 Department of Neurosurgery, Hospital Clinic de Barcelona, Universitat de Barcelona, Spain; and; 7 Department of Otolaryngology, University of Washington School of Medicine, Seattle, Washington

Minimally invasive surgery of the skull base has undergone a massive burgeoning over the last 2 decades. The catalyst to this expansion has been the acceptance of the endoscope as a means of visualizing anatomy at the depth of a narrow corridor. The development of the transorbital approach has been a natural progression that followed the rise and widespread adoption of the endonasal route. In fact, some of the areas of the skull base most amenable to the transorbital approach, namely the greater and lesser sphenoid wings and the lateral compartments of the cavernous sinus and Meckel’s cave, are mostly inaccessible by endonasal surgery. This compendium of videos demonstrates the current state of the art in endoscopic transorbital surgery and complements the recently published textbook *Endoscopic Transorbital Surgery of the Orbit, Skull Base and Brain*.^1^ The editors of this issue of *Neurosurgical Focus: Video* are mostly founding members of the recently formed Endoscopic TransOrbital Society, whose international membership grows daily.

It is important to keep in mind that orbital surgery is not a new concept. For this reason, it is critical to differentiate endoscopic transorbital surgery from existing approaches such as the lateral orbitotomy or the supraciliary (eyebrow) approach, with which the endoscopic transorbital approach has significant overlap. Indeed, as more of the orbital rim is removed, this distinction becomes further blurred. In its purest form, the endoscopic transorbital approach uses a 360° virtual corridor between the globe of the eye and the surrounding bone of the orbit, and the incision is centered in this same circumferential periorbital plane. The skin entry point for the transorbital approach is positioned nearly in the Frankfurt plane. When combined with the supine ventral patient position, this approach enables surgeons to access paramedian anterior and middle skull base targets without the need for brain retraction. Furthermore, the primary surgical vector progresses in a caudal-to-cranial direction, facilitating intradural access to both deep temporal and frontal regions, including areas medial to the optic radiations, thereby enhancing its potential future clinical applications. While the supraciliary and lateral orbitotomy approaches can lead to similar anatomical locations, the incisions are not directly adjacent to the orbit, and as a result, their approach vectors are more tangential and less orthogonal. As such, the microscope is used more frequently during the supraciliary and lateral orbitotomy approaches, whereas the pure form of the transorbital approach relies heavily on the endoscope.

Several authors have recently introduced the concept of the extended endoscopic transorbital approach, which includes removal of varying amounts of the lateral or superolateral orbital rim, which alters the approach trajectory, making the transorbital vector more tangential. These maneuvers increase the versatility of the endoscopic transorbital approach by maximizing exposure and maneuverability, which can be critical for reaching deeper targets like the petrous apex, anterior clinoid, infratemporal fossa, and posterior fossa.^2^ Likewise, the eyelid incision can be extended over the lateral canthus, which renders the trajectory even more tangential, where it begins to overlap increasingly with the lateral orbitotomy.

This issue of *Neurosurgical Focus: Video* explores the indications, technical nuances, complications, and efficacy of the endoscopic transorbital approach, as well as its variations and extensions. Authors from around the world have contributed since this novel approach has gained international attention. We hope this issue provides an introduction to some of the many procedures that can be performed with transorbital neuroendoscopic surgery.

## Disclosures

Dr. Sivakumar reported consulting fees from Stryker outside the submitted work. Dr. Di Somma reported being a consultant for Brainlab. Dr. Moe reported a patent for SpiWay licensed.
